# Influence of Non-Structural Parameters on Dual Parallel Jet Characteristics of Porous Nozzles

**DOI:** 10.3390/mi11080772

**Published:** 2020-08-14

**Authors:** Jin Zhang, Ruiqi Lv, Qifan Yang, Baolei Liu, Ying Li

**Affiliations:** 1 School of Mechanical Engineering, Yanshan University, Qinhuangdao 066004, China; zhangjin@ysu.edu.cn (J.Z.); lyuruiqi@126.com (R.L.); yqffzr@163.com (Q.Y.); liubaoleino1@163.com (B.L.); 2 School of Mechanical Engineering, Nanjing Institute of Technology, Nanjing 211167, China

**Keywords:** dual parallel jet, porous nozzle, air jet, flow field simulation

## Abstract

As an important actuator of the dual parallel jet, the porous nozzle has some non-structural parameters (such as inlet pressure, nozzle spacing ratio, etc.) which have a significant influence on energy transport, chemical combustion and pollutant generation. The research on the microfluidic state of the porous nozzle dual parallel jet, however, remains insufficient because of its microjet pattern and complex intersection process. In this paper, the authors used numerical simulation and an experimental method to clarify the influence of porous nozzles’ non-structural parameters on dual parallel jet characteristics. The results show that the inlet pressure only changes the pressure peak value on the parallel jet axis; the starting point (SP) and peak point (PP) on the parallel jet axis, which are located at *X_sp_* = 22 mm and *X_pp_* = 75 mm, respectively, are not changed; and with the increase in the nozzle spacing ratio, the merging points (MPs) on the parallel jet axis are *X_mp_* = 25 mm, 32 mm and 59 mm, respectively. The merging point and the combined point move to a farther distance and the inner deflection angle of the jet is weakened.

## 1. Introduction

The dual parallel jet is widely used in fluid energy transmission [1], atomic energy conversion [2], dynamics [3], chemical industry [4], aviation [5], and other fields. And as an important actuator of the dual parallel jet, the nozzle has a great influence on electrostatic distribution [6], three-dimensional (3D) bioprinting [7], electro-osmotic fluid flow [8], jetting performance [9], metal microcomponents picking [10], energy transport, chemical combustion and pollutant generation [11]. Thus, it is of great significance to verify the parallel jet characteristics and microfluidic state in the nozzle by means of numerical simulation and experiments. Finding a way of improving the dual parallel jet characteristics by adjusting the non-structural parameters (such as inlet pressure, nozzle spacing ratio, etc.) has become the common goal of domestic and foreign scholars [12,13,14,15].

In regard to the porous nozzle jet characteristics, Evans [16] studied the gas dispersion through porous nozzles into down-flowing liquids, and the flow characteristics of a down-flowing gas–liquid column incorporating a submerged entry porous nozzle system are considered. Okada [17] compared the behavior of liquid films and droplets in the non-equilibrium region of a downward annular mist flow under porous and central nozzle mixing methods, and displayed that the onset of large disturbance waves was influenced remarkably by the mixing method. Syamir [18] showed that porous treatments are beneficial to noise reduction, and Wei [19] obtained that the structure of the porous nozzle plays a vital role in the full utilization of energy.

Regarding the inlet pressure influence on the characteristics of the parallel jet, Fujisawa [20] studied the interaction of two parallel plane jets under two different inlet velocities, and found that the interaction of the two parallel jets weakened with the decrease in the jet velocity ratio; Khazaei [21] found that the influence of the pressure of the dual parallel jet on the extraction effect is the most significant among many factors; Tian [22] studied the influence of pressure and nozzle target distance on jet impact pressure, and the results showed that both of them can effectively improve jet efficiency; Assoudi [23] found that the velocity ratio has a significant influence on the mean velocity and turbulence characteristics of the turbulent offset jet; Krishna Chandran [24] simulated the non-isothermal jet mixing phenomena and obtained result that suggested that the jets with a unity velocity ratio showed maximum temperature fluctuations; Wang [25] investigated the flow structure nearby a rectangular cooling hole on a flat plate and proved that the asymmetric vortex system with a different velocity ratios is stable; and Kwon [26] measured the heat transfer performance of the coolers for three electronic device configurations with varied jet velocity.

Regarding the influence of the nozzle spacing ratio on the dual parallel jet characteristics, domestic and foreign researchers have found that the characteristics of dual parallel jet are related to the ratio of the nozzle axis spacing *d* to the nozzle width *w* (nozzle spacing ratio, *d/w*). Wang and Tan [27] proved that the existence of Carmen vortex led to the periodic interaction between two jets when *d/w* = 1; Mondal, Das, Guha, and Transfer [28] concluded that at 0.6 ≤ *d/w* ≤ 1.4, the peak value of jet velocity decreases with an increase in the jets’ spacing ratio; Liu et al. [11] studied the jet characteristics of rectangular nozzles, and found that the nozzle spacing ratio is linear with the merging point; Zhao and Wang [29] found that the whole flow field periodically declines at 7 ≤ *d/w* ≤ 8; and Manigandan et al. [30] studied the aerodynamic mixing characteristics of the nozzle with an identical circular exit of the spacing ratio where *d/w* = 3, and found that the mixing promoting capability decreases as the area of the elliptical throat decreases.

The above-mentioned scholars have conducted a lot of research on the porous nozzle, but most of them chose the liquid or gas–liquid mixture as the jet medium, and the research on the jet characteristics with pure gas is still insufficient. However, the jet mixing characteristics of gas play a key role in equipment cooling and jet cleaning, such as cooling of the blade passage and purging of steel surface. Thus, it is particularly important to study the performance of gas jet mixing. Moreover, the mixing characteristics of the parallel jet with porous nozzles are still unclear, and the law of the interaction between jet columns remains to be explored. The mapping relationship between the microfluidic characteristics and the operating parameters remains undiscovered, and the analysis of the influence of the operating parameters on the jet region is still insufficient. Therefore, the authors take the porous nozzle as the research subject, and numerical simulation and experiments are used to explore the influence of two non-structural parameters (the inlet pressure and nozzle spacing ratio) on the pressure and velocity distribution during parallel jet processes. The originality of this paper lies in the research on the characteristics of parallel jets with porous nozzles with gas, the finding that there are two confluences in the process, and the exploration of the influence of operation parameters on the jet flow field.

## 2. Establishment of the Simulation Model

### 2.1. Arrangement of the Dual Parallel Jet

**Figure 1:**
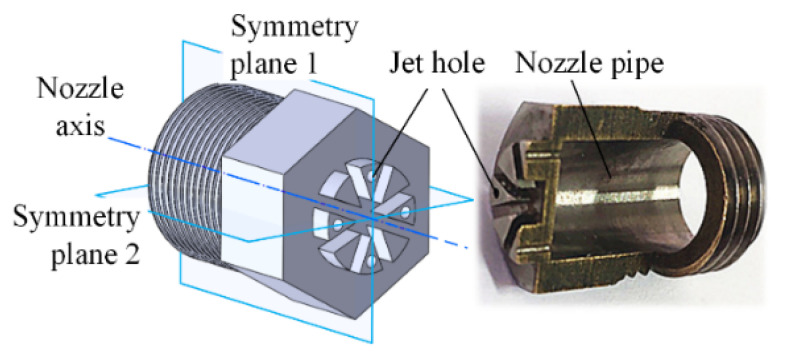
Nozzle structure.

The structure of the porous nozzle is shown in Figure 1. There are four jet holes which are symmetrically distributed at the center and eight fan-shaped bosses at the front of the nozzle to adjust the air flow state and reduce noise. The middle of the nozzle is a hexagonal structure, which facilitates the removal and installation of the nozzle; the rear thread of the nozzle is used to fix the nozzle.

The specific dimensions of the porous nozzle are shown in Table 1.

**Figure 2:**
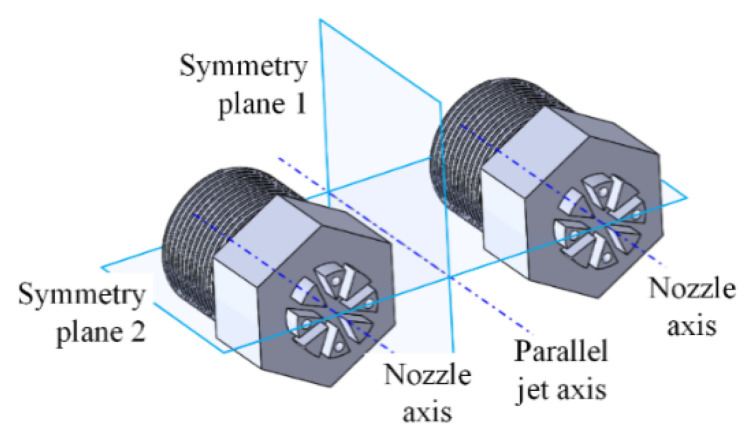
Space arrangement of the porous nozzle dual parallel jet.

In the process of the dual parallel jet, the two nozzles are parallel and symmetrically arranged along symmetry plane 1, and the jet holes adjacent to the side are located on symmetry plane 2. The dual parallel jet arrangement of the porous nozzles is shown in Figure 2.

### 2.2. Computational Domain

**Figure 3:**
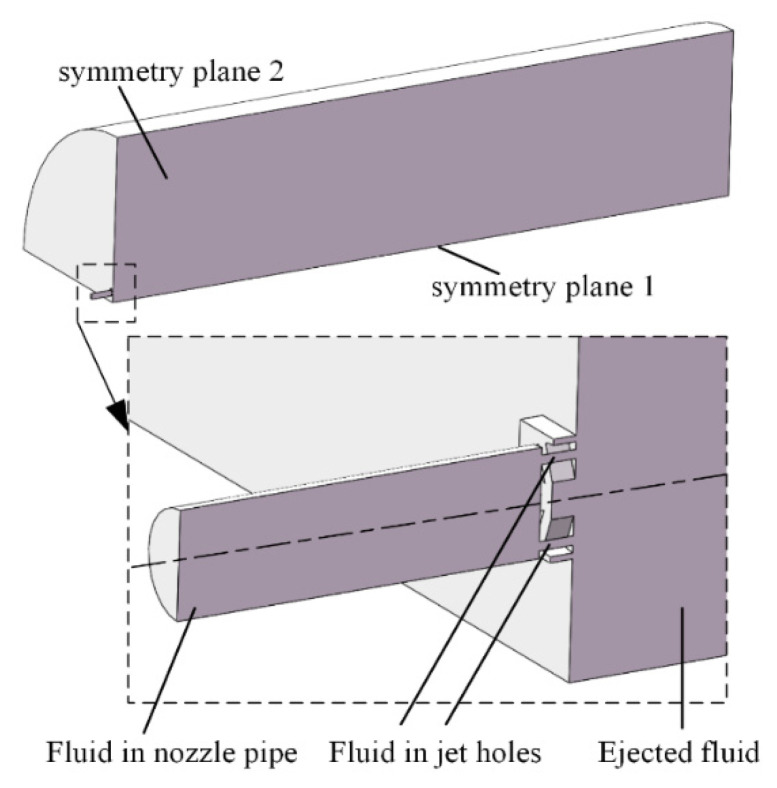
Computational domain of the dual parallel jet with porous nozzles.

In order to reduce the influence of the boundary on the jet flow field, the domain is set as a cylinder with a length of 1000 mm and a diameter of 500 mm. Considering the fact that the domain is symmetrical, a quarter of the domain is taken as the computational domain, which greatly reduces the simulation workload and shortens the research period. The computational domain for the dual parallel jet with porous nozzles is presented in Figure 3.

**Figure 4:**
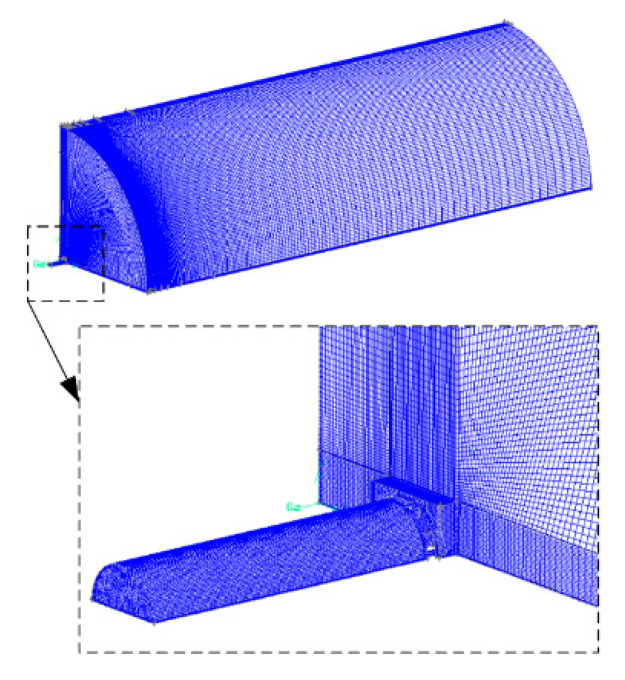
Mesh of the computational domain.

Three sets of mesh independence analysis are carried out. The grid size at the jet outlet is 0.1 mm and the grid size increases with the jet distance. The computational domain is meshed with 2,676,830 grids, as shown in Figure 4.

### 2.3. Governing Equations

The basic equations of fluid mechanics include the continuity equation, the motion equation, the energy equation and the state equation. The details are as follows:

The continuity equation:

(1) $$\frac{\partial \rho }{\partial t}+\mathrm{div}\left(\rho v\right)=0$$

The motion equation:

(2) $$\frac{\partial \left(\rho u\right)}{\partial t}+\mathrm{div}\left(\rho uv\right)=\mathrm{div}\left(\mu \mathrm{grad}u\right)-\frac{\partial p}{\partial x}+S_{u}$$

(3) $$\frac{\partial \left(\rho v\right)}{\partial t}+\mathrm{div}\left(\rho vv\right)=\mathrm{div}\left(\mu \mathrm{grad}v\right)-\frac{\partial p}{\partial y}+S_{v}$$

(4) $$\frac{\partial \left(\rho \omega \right)}{\partial t}+\mathrm{div}\left(\rho \omega v\right)=\mathrm{div}\left(\mu \mathrm{grad}\omega \right)-\frac{\partial p}{\partial z}+S_{\omega }$$

The energy equation:

(5) $$\frac{\partial \left(\rho T\right)}{\partial t}+\mathrm{div}\left(\rho vT\right)=\mathrm{div}\left(\frac{k}{c_{p}}\mathrm{grad}T\right)+\frac{S_{T}}{c_{p}}$$

The state equation:

(6) ρ=f(p,T)

There are six equations in this system, including six unknowns: fluid velocity in three directions *u*, *v*, *ω*, fluid pressure *p*, fluid temperature *T*, fluid density *ρ*. This system is closed, so it can solve the most basic hydrodynamics problems. *μ* is dynamic viscosity, also known as the momentum diffusion coefficient. $S_{u}$, $S_{v},\;S_{\omega }$, are the strain rates in different directions. $C_{p}$ is the specific heat capacity, 1030 J/(kg·°C). $S_{T}$ is the dissipation function of fluid velocity in three directions, namely, *u*, *v*, *ω*. *K* is the thermal conductivity of air, 0.024 W/(m·°C). In the initial state, the fluid velocity is 0, the fluid pressures *p* of the inlet are 0.1, 0.3 and 0.5 Mpa, respectively, and the initial gas temperature *T* is 25 °C.

However, when describing the turbulent flow state, the Reynolds stress term, which represents turbulence effect, is introduced due to the use of the Reynolds time average treatment in order to ensure that the equations are not closed. Therefore, it is necessary to determine whether the jet flow state is turbulent according to the following formula:

(7) $$Re=\frac{\rho vD}{\mu }$$

where *Re* is the Reynolds number, *D* is the diameter of the nozzle jet pipe, and *v* is the air velocity in the jet direction. By substituting the data into the above formula, it is found that the Reynolds number of the air jet of the porous nozzle is 6486, which is larger than the critical Reynolds number 2320 under the circular tube; thus, the jet state is turbulent. At this time, we are required to introduce a turbulence model to make the equations closed. The governing equation used throughout this paper is the *k-ω* equation based on the *SST* (shear stress transport) model, shown as follows:

(8) $$\frac{\mu }{\rho }=\frac{ak}{\max\left(a\omega ,SF_{2}\right)}$$

In the formula, *F*_2_ is a mixing function which constrains the wall layer as a limit number when the free shear flow does not coincide with the assumption. *S* is a fixed estimate of the strain rate. *a* = 0.031 is a constant. The biggest advantage of using the *SST* model in this paper is that the turbulent shear stress is considered, and the fluid separation at the beginning of flow and negative pressure gradient can be accurately predicted in order to ensure that the eddy viscosity is not over-predicted.

### 2.4. Boundary Conditions

**Figure 5:**
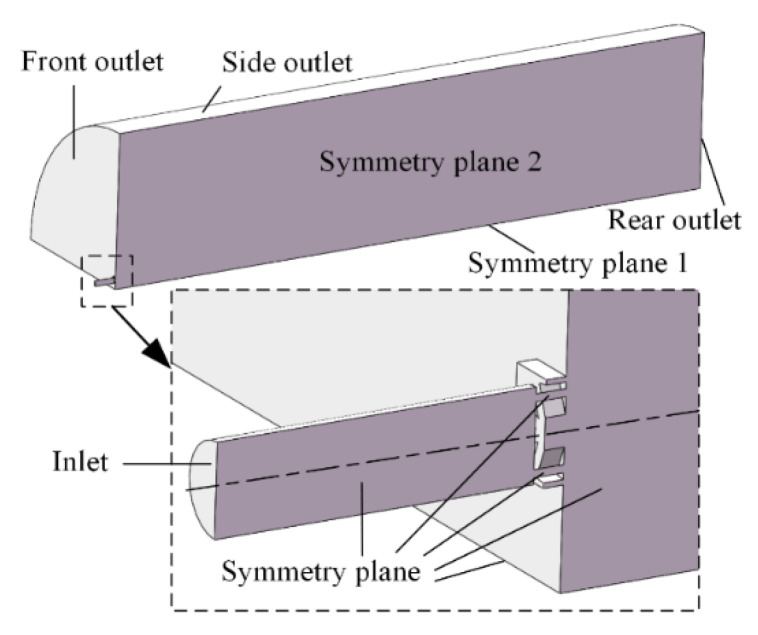
Model boundary conditions.

The boundary conditions are shown in Figure 5. The jet medium is constant air; the jet inlet is set as the pressure inlet with a gauge pressure of 0.1–0.5 MPa; the front and rear jet outlets are set as pressure outlets with a gauge pressure of 0 MPa; the symmetrical plane is set as the symmetry; and the remaining boundaries are set as the wall. The specific boundary conditions are shown in the Table 2.

## 3. Simulation Results

### 3.1. Process of the Dual Parallel Jet with Porous Nozzles

**Figure 6:**
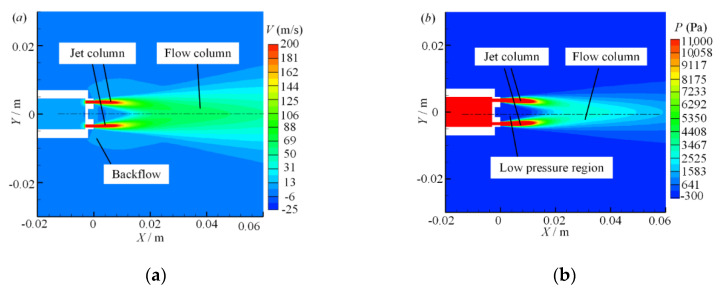
Symmetry plane 2 contours of the first confluence: (**a**) velocity contour; (**b**) pressure contour.

**Figure 7:**
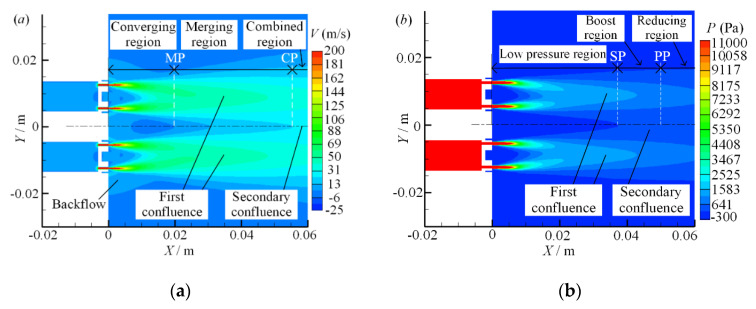
Symmetry plane 2 contours of the secondary confluence: (**a**) velocity contour; (**b**) pressure contour.

The process of the parallel jet with porous nozzles includes two confluences. The four jet holes of the single nozzle jet parallel along the nozzle axis form four independent jet columns, and the four jet columns combine into a single flow column to complete the first confluences, as shown in Figure 6. Then, the dual flow columns are combined twice to complete the secondary confluences, as shown in Figure 7.

Due to the existence of air viscous drag, when the jet air of the dual parallel jets carries the stationary air in the surrounding area forward, the air gradually mixes and merges and a low-pressure region in the middle ground is created. This low-pressure region causes backflow at the bottom of the jet.

On the velocity field of the dual parallel jet, there are three regions along the jet axis: the converging region, the merging region and the combined region [31,32,33,34]. On the parallel jet axis, the merging point (MP) is defined as the point where the backflow disappears and the axis velocity is zero, and the combined point (CP) is defined as the point where the two parallel jets disappear and merge into one and the axis velocity is at its maximum. The converging region spans from the jet outlet to the merging point (MP), between the merging point (MP) and the combined point (CP) is the merging region, and the combined region is after the combined point (CP).

For the division of the pressure region in the parallel jet process, there are also three regions along the jet direction that are defined [35,36]: the low-pressure region, the boosting region and the reducing region. On the parallel jet axis, the starting point (SP) is defined as the rapid rising point of the pressure value from zero, and the peak point (PP) is defined as the highest point of pressure on the axis. The low pressure region spans from the jet outlet of the nozzle to the starting point (SP), the boosting region spans from the starting point (SP) to the peak point (PP), and the reducing region is after the peak point (PP).

### 3.2. Influence of Inlet Pressure on Parallel Jet Characteristics

**Figure 8:**
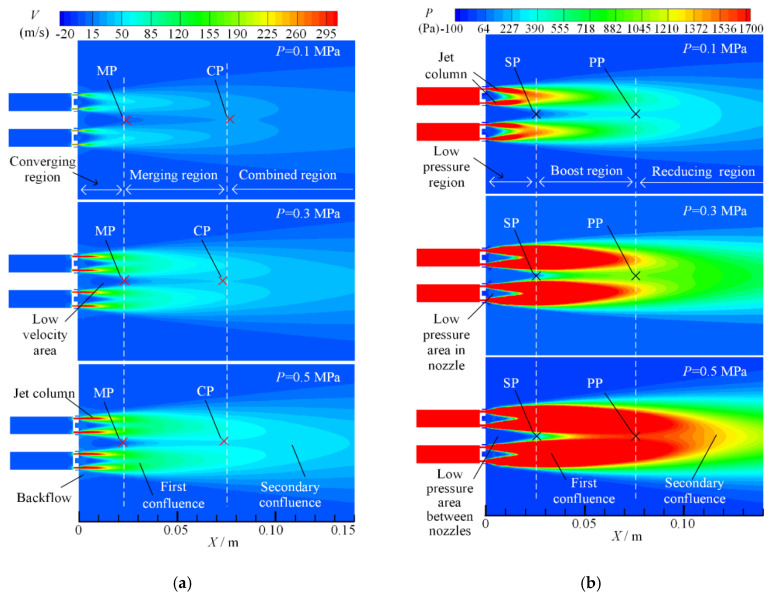
Symmetry plane 2 velocity contours with different inlet pressures: (**a**) velocity contour; (**b**) pressure contour.

When the nozzle spacing ratio (*d/w* = 3) is fixed and the inlet pressure *P* is 0.1 MPa, 0.3 MPa and 0.5 MPa, respectively, the velocity and pressure contours of symmetry plane 2 are as shown in Figure 8.

It can be seen from Figure 8a that as the inlet pressure increases, the jet column velocity increases after the air exits from the jet holes. Thus, the first and secondary confluence effects are more significant, the overall flow region of dual parallel jet is enlarged, and the overall flow velocity is increased, but the low velocity area between the nozzles shrinks and the backflow is more significant. From the combined velocity distribution and variation trend on the parallel jet axis, it can be observed that under different inlet pressures, the merging point (MP) is distributed at the same point of *X* = 22 mm, and the position of the combined point (CP) is located at the same point of *X* = 75 mm. The merging point (MP) and the combined point (CP) on the parallel jet axis do not change with the increase in inlet pressure, which also means that the division of the converging region, merging region and combined region does not change.

Therefore, in the process of the dual parallel jet with porous nozzles, the inlet pressure only affects the value of velocity. The greater the inlet pressure, the larger the velocity of parallel jet and the greater the peak value of velocity on the parallel jet axis. However, in the process of the dual parallel jet, the velocity-change rule remains; that is, it converges first, then merges, and combined finally. Additionally the axial ranges of the converging region and the merging region are fixed, always kept within the range of *X*~(0 mm, 22 mm) and *X*~(22 mm, 75 mm) in order to ensure that the mixing fluctuation range of the parallel jet is certain.

It can be seen from Figure 8b that with the increase in inlet pressure, the axial and radial ranges of the jet column expand, which leads to the expansion of the pressure impact region of the first and second confluences, and the overall flow region is widened. The low-pressure areas between the nozzles and in the nozzles are narrowed, but the starting point (SP) and the peak point (PP), which are located at *X* = 22 mm and *X* = 75 mm, respectively, on the parallel jet axis are not changed at the same point as the velocity-merging point (MP) and velocity-combined point (CP). Moreover, the diversion of the low pressure region, the boosting region and the reducing region does not change as well with the increase in the inlet pressure. The velocity region corresponds to the pressure region.

### 3.3. Influence of the Nozzle Spacing Ratio on the Characteristics of the Parallel Jet 

**Figure 9:**
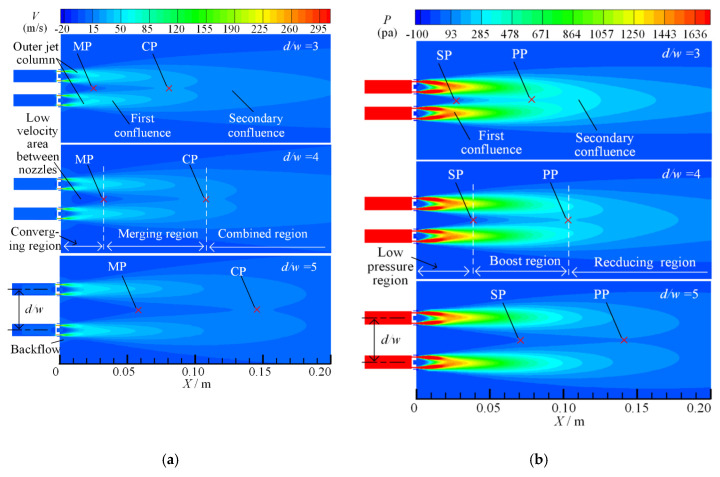
Symmetry plane 2 contours with different nozzle spacing ratios: (**a**) velocity contours; (**b**) pressure contours.

When the inlet pressure (*P* = 0.1 MPa) is fixed and the nozzle spacing *d/w* ratio is 3, 4 and 5, respectively, the velocity and pressure contours on symmetrical plane 2 of the dual parallel jet are as shown in Figure 9.

It can be seen from Figure 9a that in the process of the first confluence, when the nozzle spacing *d/w* ratio is 3, the outer jet column deflects significantly to the inner side under the influence of the whole parallel jet, and its axial jet distance is longer than that of the inner jet column; when the nozzle spacing ratio is *d/w* = 4, the inner deflection angle of the outer jet column decreases; and when the nozzle spacing ratio is *d/w* = 5, the inner deflection angle of the outer jet column is significantly smaller than that of the ratio when *d/w* = 3, and the outer jet column and the inner jet column on symmetry plane 2 are nearly symmetrical. Therefore, with the increase in the nozzle spacing ratio, the influence of the whole dual parallel jet on the first confluence decreases.

In the secondary confluence process, when the nozzle spacing ratios are *d/w* = 3, 4 and 5, the merging points (MPs) on the parallel jet axis are 25 mm, 32 mm and 59 mm, respectively, and the combined points (CPs) on the parallel jet axis are 80 mm, 108 mm and 135 mm, respectively. The merging point (MP) and the combined point (CP) move backward gradually, and the ranges of converging region, merging region and combined region expand correspondingly. The backflow phenomenon weakens, and the low velocity area between the nozzles is grows. Thus, the merging region is extended, which means that the jet stability is reduced and the dual parallel jet merges at a greater distance in the axial direction.

It can be seen from Figure 9b that when the nozzle spacing *d/w* ratio is 3, 4 and 5, the pressure starting point (SP) on the parallel jet axis is 27 mm, 38 mm and 69 mm, respectively, and the pressure peak point (PP) is 80 mm, 102 mm and 132 mm, respectively. The changes in the pressure starting point (SP) and peak point (PP) are consistent with the changes in the velocity-merging point (MP) and the combined point (CP), and they all move to a farther distance with the increase in the nozzle spacing ratio. The overall flow region expands along the axial direction and longitudinal direction. The low-pressure region, the boosting region and the reducing region are expanded, and the dual parallel jet converges at a greater distance; the secondary confluence has a smaller impact on the primary confluence.

## 4. Experimental Result and Discussion

**Figure 10:**
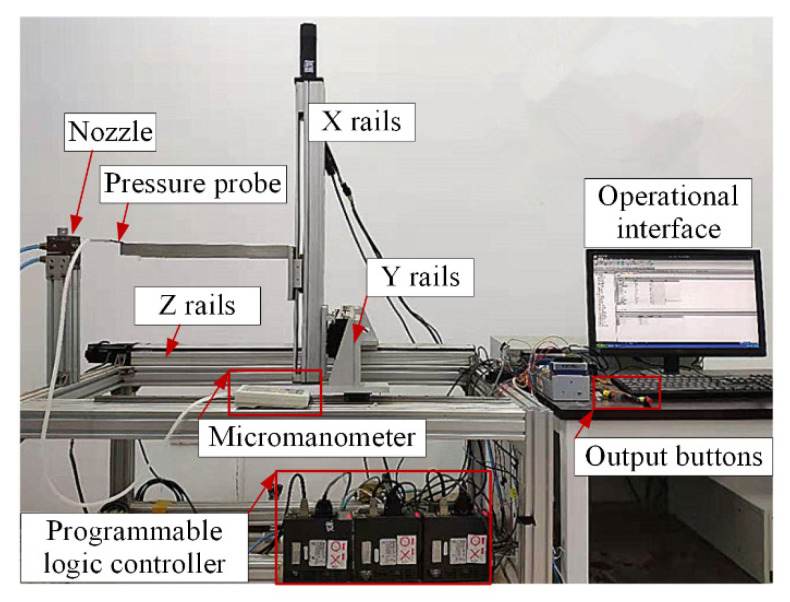
Experimental test system.

In this experiment, the pressure value on the parallel jet axis was collected and compared with the simulation results. As shown in Figure 10, the experimental test system is mainly composed of an operational interface, output buttons, a programmable logic controller, three-degree-of-freedom nozzle-moving rails, porous nozzles, a pressure probe and an air compressor.

**Figure 11:**
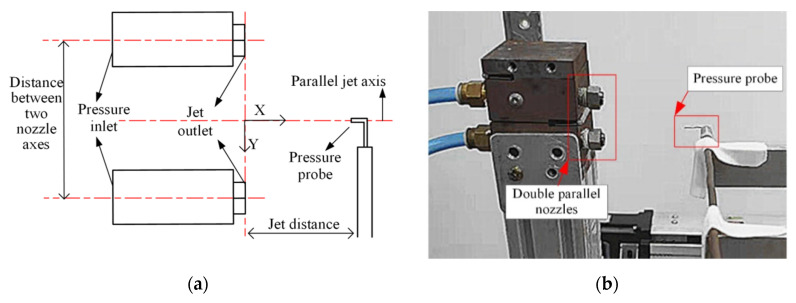
Experimental scheme: (**a**) experimental test principle; (**b**) experimental arrangement.

The arrangement of the nozzles in the experiment is shown in Figure 11.

The working parameters of the micromanometer are shown in Table 3.

**Figure 12:**
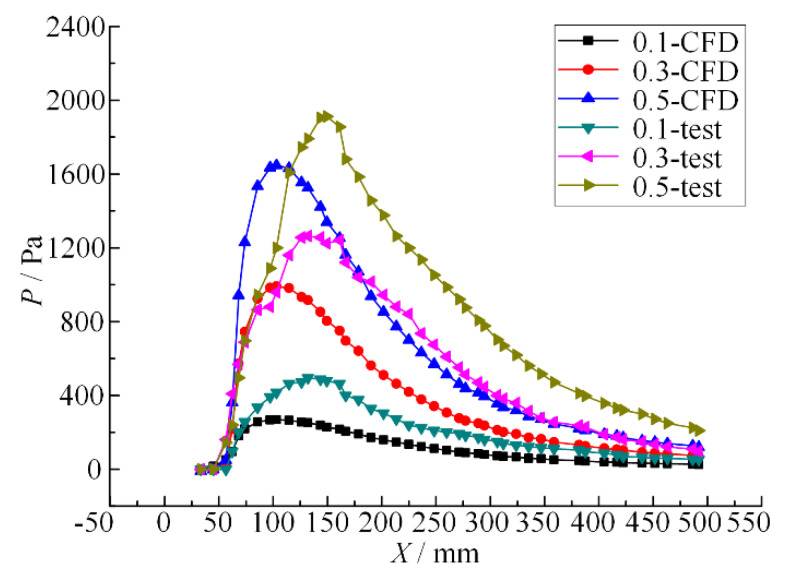
Pressure curves of the dual parallel jet axis with different inlet pressures.

When the nozzle spacing *d/w* ratio is 3 and the inlet pressure is 0.1 MPa, 0.3 MPa and 0.5 MPa, respectively, the pressure of the dual parallel jet axis is as shown in Figure 12.

It can be seen from Figure 12 that when the nozzle spacing ratio is fixed, the inlet pressure is different and the pressure change in the parallel jet axis shows a similar trend of a slow decline after a rapid rise from zero pressure. With the increase in inlet pressure, (1) the rate of pressure change increases, (2) the peak pressure increases, and (3) the starting point (SP) and peak point (PP) do not change. The results show that the pressure value and change rate of the parallel jet axis are related to the inlet pressure, and at the same point on the parallel jet axis, the larger the inlet pressure, the higher the pressure value and the greater the pressure change rate. However, the starting point (SP) and peak point (PP) do not change with the increase in inlet pressure, and the division among the low pressure region, boosting region and reducing region also has no effect. Thus, the inlet pressure can only affect the peak value of the parallel jet with porous nozzles and the energy conversion of the confluence will increase accordingly, while the completed merging point does not change and the peak value always appears at the same point. Accordingly, the optimum distance of nozzle jet cleaning does not change with the increase in jet pressure.

**Figure 13:**
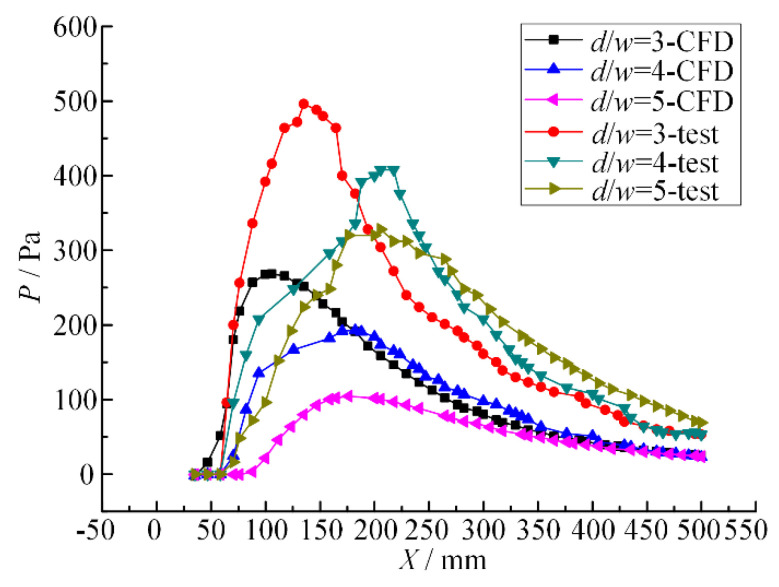
Pressure curves of the dual parallel jet axis with different nozzle pitch ratios.

When the inlet pressure is 0.1 MPa and the nozzle spacing *d/w* ratio is 3, 4 and 5, respectively, the pressure change curves of the dual parallel jet axis are as shown in Figure 13.

It can be seen from Figure 13 that under certain inlet pressure, the nozzle spacing ratio is different, and the pressure change trends of the experiment and simulation on the parallel jet axis are the same—both of them rise rapidly from zero pressure to their peak value, decline slowly, and finally merge and decay. With the increase in the nozzle spacing ratio, (1) the starting point (SP) and the peak point (PP) move backward; (2) the change rate of pressure becomes smaller and both rise and fall speeds decrease; and (3) the peak value of pressure decreases.

This indicates that as the nozzle spacing ratio increases in the dual parallel jet process, the pressure starting points (SP) and the peak point (PP) move backwards, causing the boosting region and the reducing zone to move backwards accordingly and the dual parallel jet to merge further away from the jet outlet. The pressure change rate of the boosting region is reduced, the pressure rise slows down, the axial length of the boosting region increases, the parallel jets merge further away, the intensity of energy conversion decreases in the process of parallel jet merging, the jet flow becomes more unstable, the pressure peak decreases, and the jet impact effect weakens.

Liu [24] fitted the equation between *X_pp_* and *d/w* by linear regression, and the peak point *X_pp_* can be forecasted through the equation. The equation is as follows:

(9) $$X_{pp}=[3.79(\frac{d}{w})+2.02]w$$

When the *d/w* ratio is 3, 4 and 5, respectively, the theoretical and average value of peak point *X_pp_* are as shown in Table 4. The errors between the average value and the theoretical value are 0.38%, 1.07% and 4.70%, respectively.

The error between the average value in this paper and Liu’s theoretical prediction value is small, so the analysis result is credible. The error increases with the increase in the nozzle spacing ratio, because the stability of the parallel jet decreases and the mixing area of the jet extends along the axis, causing the measurement accuracy to decrease.

## 5. Conclusions

(1) The process of the dual parallel jet with porous nozzles contains two confluences: (1) the single nozzle jet columns merge into a flow column; and (2) the two flow columns merge into the whole flow region, and then the flow recedes gradually.

(2) When the inlet pressure in the dual parallel jet is 0.1 MPa, 0.3 MPa, 0.5 MPa, the pressure and velocity values and their change rate increase, and the overall jet region expands. However, the velocity merging point (MP) and the combined point (CP) are consistent, always at *X_mp_* = 22 mm, *X_cp_* = 75 mm; the pressure starting point (SP) and the peak point (PP) are maintained, always at *X_sp_* = 22 mm and *X_pp_* = 75 mm. The pressure and velocity flow fields are consistent.

(3) When the nozzle spacing ratio increases from 3 to 5, the influence of the overall dual parallel jet on the outer jet column weakens. The pressure starting points (SP) on the parallel jet axis are *X_sp_* = 27 mm, 38 mm and 69 mm, respectively, and the peak points PP are *X_pp_* = 80 mm, 102 mm and 132 mm, respectively. The two parallel jets merge further away from the jet outlet, and the pressure-boosting region and -reducing region also move further away from the outlet correspondingly.

## Figures and Tables

**Table 1:** Nozzle structure parameters.

Parameter	Value
diameter of the jet port *D*_1_	1 mm
internal diameter of the nozzle pipe *D*_2_	9 mm
nominal diameter of the thread *D*_3_	13 mm
height of the sector boss *H*_1_	2 mm
Height of the hexagon *H*_2_	8 mm
distance between the opposite sides of the hexagon *H*_3_	14 mm
distance between the opposite jet holes *w*	6 mm
distance between the two nozzle axes *d*	18/24/30 mm

**Table 2:** Boundary conditions.

Parameter	Value
medium	Constant air
pressure inlet	0.1/0.3/0.5 MPa
pressure outlets	0 Mpa
symmetrical plane	Symmetry
other boundaries	Wall

**Table 3:** Working parameters of the micromanometer.

Parameter	Value
range	0–±8000 Pa
accuracy class	0.5 FS
operation temperature	25 °C

**Table 4:** Error statistics of peak point *X_pp_*.

Spacing Ratio *d/w*	Theoretical Value	Average Value	Error
3	80.3 mm	80 mm	0.38%
4	103.1 mm	102 mm	1.07%
5	125.8 mm	132 mm	4.70%
